# Insights from the evaluations of the NIH Centers for Accelerated Innovation and Research Evaluation and Commercialization Hubs programs

**DOI:** 10.1017/cts.2021.878

**Published:** 2021-11-22

**Authors:** Benjamin J. Anderson, Olena Leonchuk, Alan C. O’Connor, Brooke K. Shaw, Amanda C. Walsh

**Affiliations:** Center for Applied Economics and Strategy, RTI International, Research Triangle Park, North Carolina, USA

**Keywords:** Academic entrepreneurship, biomedical innovation, proof-of-concept, accelerator, evaluation, commercialization, technology development, SBIR/STTR

## Abstract

**Background:**

The National Institutes of Health launched the NIH Centers for Accelerated Innovation and the Research Evaluation and Commercialization Hubs programs to develop approaches and strategies to promote academic entrepreneurship and translate research discoveries into products and tools to help patients. The two programs collectively funded 11 sites at individual research institutions or consortia of institutions around the United States. Sites provided funding, project management, and coaching to funded investigators and commercialization education programs open to their research communities.

**Methods:**

We implemented an evaluation program that included longitudinal tracking of funded technology development projects and commercialization outcomes; interviews with site teams, funded investigators, and relevant institutional and innovation ecosystem stakeholders and analysis and review of administrative data.

**Results:**

As of May 2021, interim results for 366 funded projects show that technologies have received nearly $1.7 billion in follow-on funding to-date. There were 88 start-ups formed, a 40% Small Business Innovation Research/Small Business Technology Transfer application success rate, and 17 licenses with small and large businesses. Twelve technologies are currently in clinical testing and three are on the market.

**Conclusions:**

Best practices used by the sites included leadership teams using milestone-based project management, external advisory boards that evaluated funding applications for commercial merit as well as scientific, sustained engagement with the academic community about commercialization in an effort to shift attitudes about commercialization, application processes synced with education programs, and the provision of project managers with private-sector product development expertise to coach funded investigators.

## Introduction

Much has been written about the challenges of translating biomedical discoveries from academic research into products and research tools that, per the mission of the National Institutes of Health (NIH), enhance health, lengthen life, and reduce illness and disability. Culprits are long and costly innovation cycles fraught with risk [1-4], sparse funding for proof-of-concept research [5], limited knowledge, and hands-on experience among academic researchers about how to move discoveries into the translational pipeline [6-8], insufficient resources at research institutions for assisting with the same [9], and an academic rewards system that prioritizes publications and novelty [10]. NIH’s Small Business Innovation Research (SBIR) and Small Business Technology Transfer (STTR) programs provide over one billion dollars each year for early-stage biomedical research and development [11], but only small businesses are eligible to apply. Academic investigators must develop sufficient evidence of the viability of their technology to take the nontrivial steps of launching a start-up company, or to convince an existing small business to license an early-stage product candidate. That leaves a lot of daylight between a promising discovery and the United States’ signature early-stage commercialization support program.

In 2010, two NIH National Heart, Lung, and Blood Institute (NHLBI) working groups were charged with improving the translation of NHLBI’s investments in its research and SBIR/STTR programs into commercial applications that improve health [12]. Two outcomes from the team’s work are relevant to this study: the creation of an office within NHLBI to support small business and translational programs and the creation of the NIH Centers for Accelerated Innovations (NCAI) program that this office would lead. The NCAI program would be a proof-of-concept program to fill the gap between discovery phase research and eligibility for the SBIR/STTR program [13,14].

The NCAI program was launched in September 2013. The program’s aim was to fund three consortia (“sites”) of at least five institutions around the country to develop and pilot solutions to address the barriers described above and accelerate the development and market-readiness of promising technologies, with the expectation that lessons and insights from the sites would then be broadly disseminated to and adopted by the academic community. NHLBI intentionally sought regional sites with multiple partner institutions to foster interinstitutional collaboration; ensure a pipeline of “center-ready” technologies in NHLBI’s heart, lung, blood, and sleep mission; and leverage resources and strategies within each site’s innovation ecosystem.

In 2015, NIH launched the Research Evaluation and Commercialization Hubs (REACH) program through the Phase 0 Proof of Concept Partnership Pilot Program authority granted in the 2011 SBIR/STTR Reauthorization Act. Like NCAI, REACH selected regional sites to develop pilot approaches and build capacity, but unlike NCAI, it covered the entire NIH mission and was not limited to the NHLBI’s heart, lung, blood, and sleep focus. It also selected sites outside of comparatively rich ecosystems such as those in Boston and California and granted them autonomy to establish themselves to best meet the needs of their investigator community. REACH has funded two cohorts of sites: three in 2015 and five in 2019.

This study presents outcomes data for technologies supported by the NCAI and REACH programs (as of May 2021) and describes practices that the NCAI and REACH sites employed to support their investigator communities that were particularly effective. NHLBI and NIH invested in a long-term evaluation program to monitor and learn from sites’ experiences resulting in a wide range of metrics and instruments designed and data collected that are summarized in Methods Overview section. That evaluation continues (and additional papers are forthcoming), but this study is an opportunity to disseminate information on observed progress to-date and some overarching lessons learned.

## Background on the NCAI and Reach Programs

NCAI and REACH are focused on furthering academic entrepreneurship and demystifying the process of translating a discovery into the product development pipeline. They are complementary programs with much in common, but there are also distinct differences. This section provides an overview.

In an effort to promote experimentation and development of novel approaches, the funding opportunity for sites under each program articulated NIH’s expectations but permitted teams to design operating plans and programs that sites believed best met the needs of their investigator community. Sites were expected to

- provide entrepreneurship training and skills development programs,
- develop a pipeline of potential projects and solicit funding applications,
- provide technology development funding for projects selected based on their commercial and scientific merit,
- have external advisory boards to guide the site and review applications,
- develop and implement market-focused project management oversight and milestone-based decision-making processes,
- leverage resources from Clinical and Translational Science Awards and local innovation ecosystems, and
- further NIH goals around culture change with respect to research translation and commercialization.^1^

The NIH program officers for both NCAI and REACH were within NHLBI, which in addition to leading NCAI administered REACH on behalf of the NIH. REACH and NCAI sites share insights and learnings with one another through annual meetings, working groups, and joint web meetings coordinated by NIH. Thus, there were organizational structures that facilitated collaboration at the NIH, program, and site levels.

Given these similarities, NCAI and REACH shared some processes. Most notable among these was a NIH-led committee known as the Technology Guidance Committee (TGC).^2^ The TGC was composed of product development experts from the NIH, the Food and Drug Administration, the Centers for Medicare and Medicaid Services, the US Patent and Trademark Office, the National Science Foundation, and Kaiser Permanente. Each site had external advisory boards that reviewed funding applications and made selections. The TGC was designed to review short-listed applications prior to final selection to provide feedback on relevant scientific, intellectual property, regulatory, and reimbursement issues. This feedback was then available to applicants to support their learning journey and technology development plans.

Key differences between NCAI and REACH include overall funding levels, award periods, mission space, locations, and specifications about the minimum number of institutions that could comprise a site. Below we describe these differences.

### NIH Centers for Accelerated Innovations Program

Launched in 2013, the NCAI program is funded by NHLBI and focused on NHLBI’s heart, lung, blood, and sleep mission. NHLBI awarded three sites, each with one prime institution and at least four other member institutions. The sites are (Table 1): the Boston Biomedical Innovation Center (B-BIC), the NIH Center for Accelerated Innovations at the Cleveland Clinic (NCAI-CC), and the University of California Center for Accelerated Innovation (UC CAI).

**Table 1.** NCAI and REACH sites and institutions

Site	Institution(s)
NIH Centers for Accelerated Innovation (NCAI)	
Boston Biomedical Innovation Center (B-BIC)	Brigham and Women’s Hospital, Harvard Medical School, Massachusetts General Hospital, Partners Healthcare, Beth Israel Deaconess Medical Center, Boston Children’s Hospital, Boston Medical Center, Boston University, Boston VA Healthcare System, Brown University, Draper Laboratory, Forsyth Institute, Maine Medical Center, Massachusetts Institute of Technology, Northeastern University, Tufts University
NIH Center for Accelerated Innovations at the Cleveland Clinic (NCAI-CC)	Cleveland Clinic, Case Western Reserve University, Cincinnati Children’s Hospital, Ohio State University, Northwestern University, University of Cincinnati, University of Michigan
University of California Center for Accelerated Innovation (UC CAI)	University of California (UC) Los Angeles, UC, Davis, UC Irvine, UC San Diego, and UC San Francisco
Research Evaluation and Commercialization Hubs (REACH), 2015	
Long Island Bioscience Hub (LIBH)	Stony Brook University, Brookhaven National Laboratory, Cold Spring Harbor Laboratory, Feinstein Institute
Minnesota REACH (MN-REACH)	University of Minnesota
University of Louisville Expediting Commercialization, Innovation, Translation, and Entrepreneurship (ExCITE)	University of Louisville
Research Evaluation and Commercialization Hubs (REACH), 2019	
SPARK REACH	University of Colorado Anschutz Medical Campus
Rutgers Health Advance	Rutgers University
Kentucky Network for Innovation & Commercialization (KYNETIC)	University of Kentucky, University of Louisville, Eastern Kentucky University, Kentucky Community and Technical College System, Kentucky State University, Morehead State University, Murray State University, Northern Kentucky University, Western Kentucky University
Midwest Biomedical Accelerator Consortium (MBArC)	University of Missouri-Columbia, University of Kansas Medical Center, Emporia State University, Fort Hays State University, Langston University, Kansas State University, Missouri University of Science and Technology, Oklahoma University, Pittsburg State University, University of Missouri-Kansas City, University of Missouri-St. Louis, University of Nebraska Medical Center, University of North Dakota, University of South Dakota Washburn University, Wichita State University
Washington Entrepreneurial Research & Commercialization Hub (WE-REACH)	University of Washington

Institutions appearing first lead the site, with all members listed alphabetically thereafter.

There was heterogeneity across the three sites with respect to funding award sizes (e.g., $50,000 to as much as $400,000) and types (small pilot projects of short duration or 1- to 2-year projects), funding solicitation processes, project management approaches, and the distribution of administrative and management effort and responsibility between the prime site and their member institutions [15]. Over time, B-BIC added three additional institutions and the NCAI-CC added two. Thus, there are a total of 29 institutions in the NCAI program.

NHLBI committed $31.5 million to the program for a 7-year period of performance [16]. B-BIC, NCAI-CC, and UC CAI also assembled a combined $23 million in institutional support and partnerships. The program was extended 1 year to permit underway projects to complete and is anticipated to conclude in 2022.^3^ No further NCAI sites are expected because NHLBI has consolidated its translational support programs into one national program called Catalyze.^4^

### Research Evaluation and Commercialization Hubs Program

The REACH program is the Phase 0 Proof-of-Concept Partnership pilot program launched in accordance with Section 5127 of the 2011 SBIR/STTR Reauthorization Act. The Act specifically called for development of proof-of-concept programs to pilot approaches for supporting academic investigators in commercializing technologies. NIH used the program as an opportunity to pilot approaches and build academic entrepreneurship capacity in a variety of geographies across the country.

Each REACH site received a smaller award amount ($1 million per year) than NCAI sites, had a shorter period of performance (3 or 4 years), was not prescribed how many institutions could comprise a site, and could support any technologies within the NIH mission space.

There are two cohorts of REACH sites. REACH 2015 includes the Long Island Bioscience Hub (LIBH) led by Stony Brook University, MN-REACH at the University of Minnesota, and the University of Louisville Expediting Commercialization, Innovation, Translation, and Entrepreneurship (ExCITE) program. NIH committed $9 million to REACH, with each site receiving $1 million per year. The three sites matched the NIH award 1:1 with institutional support and partnerships. There were six institutions in the program because LIBH had four partners. REACH 2015 concluded in 2021 after being extended to support projects funded late in the original period of performance.

Section 5127 was reauthorized, permitting the launch of a second cohort of REACH sites. REACH 2019 includes five sites: Rutgers HealthAdvance at Rutgers University, SPARK REACH at the University of Colorado Anschutz Medical Campus, the Kentucky Network for Innovation & Commercialization (KYNETIC) led by the University of Kentucky, the Midwest Biomedical Accelerator Program (MBArC) led by the University of Missouri, Columbia, and WE-REACH at the University of Washington. NIH committed $20 million to the program, with each site receiving $1 million per year for 4 years. Each site matched the NIH award 1:1 with institutional support and partnerships.

There are many more institutions in REACH 2019 than REACH 2015 because two sites are consortia. KYNETIC covers all public institutions of higher education in Kentucky, including its research universities, regional universities, and community and technical college system. MBArC has member institutions in six states: Missouri, Kansas, Nebraska, Oklahoma, North Dakota, and South Dakota. In total, there are 43 institutions in REACH 2019.

## Methods Overview

The authors are independent evaluators engaged by NHLBI and NIH to evaluate the NCAI and REACH programs. A common goal of the evaluations is to identify best practices and lessons learned through on-going observation and analysis of the design, processes, and outcomes for each of the 11 NCAI, REACH 2015, and REACH 2019 sites. We developed and implemented an evaluation program that included longitudinal tracking of funded technology development projects and associated commercialization outcomes; interviews with site teams, funded investigators, and relevant institutional and innovation ecosystem stakeholders; periodic surveys of program participants about commercialization knowledge, attitudes, and beliefs; and analysis and review of administrative data.^5^ The evaluation program was launched in 2015 and continues as of this writing. More nuanced discussion procedures relevant for this paper accompanies the review of our commercialization outcomes and high-level observations of best practices and lessons learned from the two programs.

## Technology Development Outcomes

**Fig. 1.**
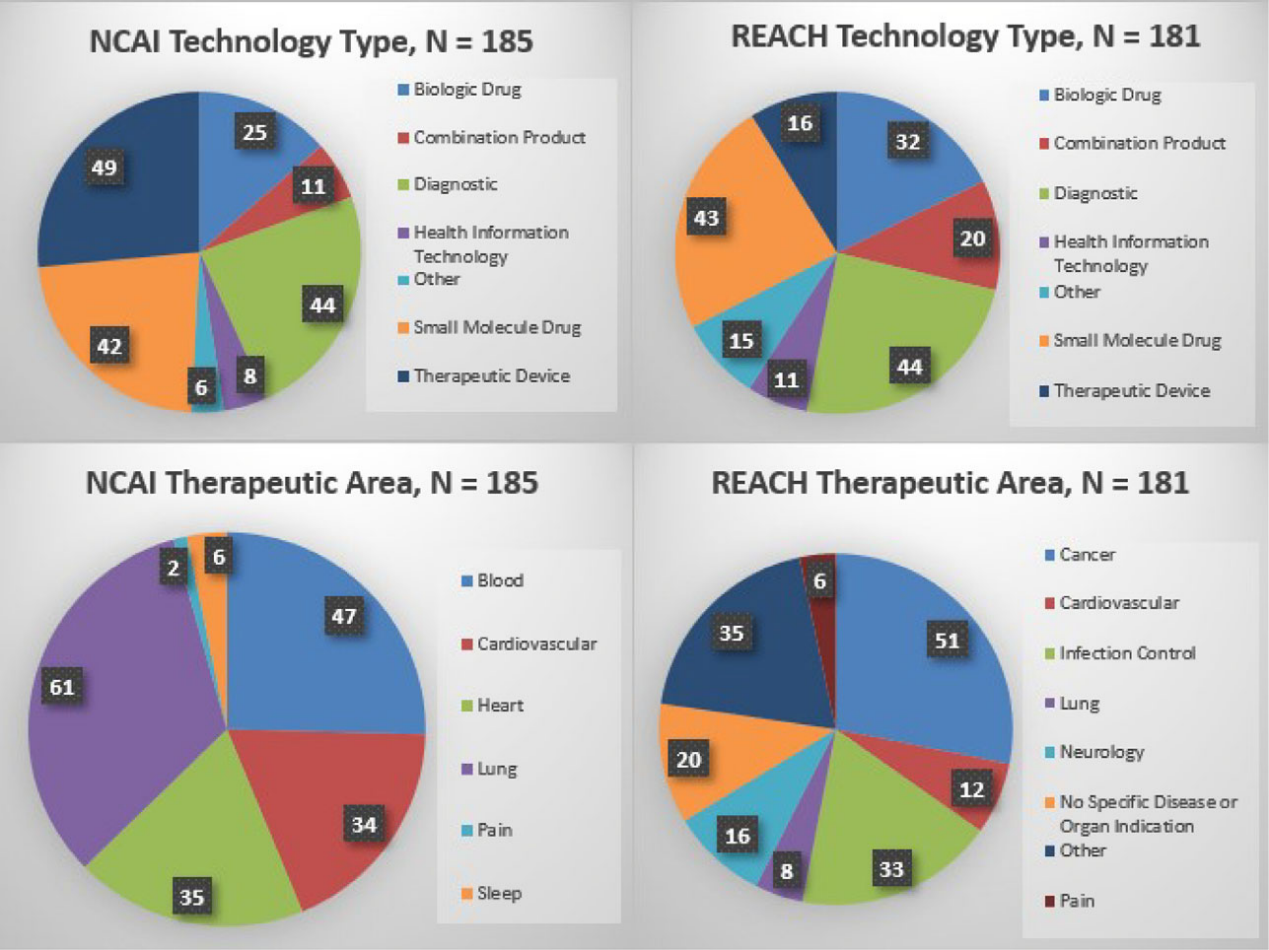
NCAI and REACH project portfolio, by technology type and therapeutic area. *Note*: NCAI, NIH Centers for Accelerated Innovation; REACH, Research Evaluation and Commercialization Hubs.

As of May 2021, NCAI and REACH sites have received a total of 1738 applications for technology-development support and ultimately funded a portfolio of 366 projects that is diverse in terms of technology type and therapeutic area (Fig. 1). NCAI funded 185 projects, of which 161 have been completed (i.e., concluded the funding award period) and 24 are still underway. REACH funded 181 projects, with 138 completed projects being mostly with REACH 2015 and 43 underway projects being with REACH 2019. REACH 2019 will continue to award new projects through 2023. This section provides a high-level overview of technology development outcomes to-date.

### Data Collection Approach

Technology development outcome measures should be considered in the context of the typical timelines required to transition early-stage, preclinical technologies from academic settings to the market. A meta-analysis identified an average time lag from discovery to clinical practice of 17 years, which is likely even longer if considering only academic-based discoveries [17]. Accordingly, we closely tracked outcomes, including funding secured for further technology development, investigators’ participation in skills development programs, start-up companies, SBIR/STTR applications and awards, technology readiness levels,^6^ and regulatory milestones and approvals (Table 2). These measures signal progression of the technologies toward the marketplace, as indicated by interest by external investors, small business formation and growth, and commercial interest in the technology. As will be discussed below, some of the NCAI and REACH technologies have indeed transitioned from the lab to patients within the short time since their awards, either into clinical studies or the marketplace.

**Table 2.** Definitions of commercialization outcome metrics

Outcome	Description
Follow-on funding	Measures the dollar amount of outside investment attracted by the technologies in the NCAI and REACH portfolio after the date of the NCAI or REACH award and provides a signal of interest and perceived value from outside entities
Start-up companies	Measures whether a company has been formed by the investigator specifically for the purpose of progressing the NCAI- or REACH-funded technology and provides a signal of technological progression and commercial viability (because of the financial and time investment associated with company formation)
Licensing and option-to-license agreements not associated with a start-up	Measures whether a technology not associated with a start-up company has licensed its technology or has signed an option to license its technology to an outside entity and provides a signal of commercial viability as assessed by outside entities
SBIR and STTR applications and awards	Measures whether the start-up company associated with the technology has applied for and been awarded an SBIR or STTR grant and provides a signal of advancement beyond early-stage research and higher probability of advancement beyond the “valley of death” stage
Technologies in clinical testing	Measures whether a technology has initiated testing on human subjects, irrespective of the technology’s regulatory path to market. This can include clinical testing conducted outside the United States
Technologies in the market	Measures whether a technology has reached the marketplace, irrespective of the technology’s regulatory path to market. This can include technologies that do not require regulatory market approval and technologies marketed outside the United States

NCAI, NIH Centers for Accelerated Innovation; REACH, Research Evaluation and Commercialization Hubs; SBIR, Small Business Innovation Research; STTR, Small Business Technology Transfer Research.

Successful analysis of outcomes relies on the quality of the data collected. We developed systematic processes and tools to enable quantitative analyses, including a web-based platform for efficient data collection and quality assurance systems for data coming from geographically dispersed sites. Data on the projects’ outcomes are collected via a semiannual review of all technologies, which is typically completed by the sites’ project managers, reviewed by the leadership of each site, and then validated by our team. The assessment includes two parts: project background information, which is completed only once; and technology development outcomes, which are reported during each review cycle. Project start dates are staggered because each site held solicited applications at least once per year, from the launch of the NCAI program to present. Longitudinal tracking over many years is an important component of the evaluation program because the timescale for commercialization for any given project is long. Projects that have been newly funded since the previous review cycle report on their characteristics and baseline status, as well as any outcomes that have transpired since the start of the project up to the time of this initial reporting. For projects that have already been captured in a previous cycle, the follow-up reviews simply elicit those new outcomes that have transpired since the previous cycle. Our team processed and validated outcome data using a variety of public and private databases for funding, clinical databases, and other information resources. However, we note that there can be some under or over reporting because of the difficulty of tracking these types of outcomes.

### Results, as of May 2021

Table 3 reports high-level outcomes for NCAI and REACH sites as of May 2021. The NCAI program’s first awards were made in mid-2014, the REACH 2015 program in late 2015, and the REACH 2019 program in spring 2020. The early-stage technology development projects funded by these programs have a period of performance of 1–2 years. The oldest NCAI and REACH 2015 projects are about 7 and 5 years post-award, respectively, at the time of writing and many projects are still underway. With only 1 year of data, it is too soon to draw any conclusions about REACH 2019. However, with seven start-ups and multiple SBIR/STTR applications, there is an observable progress, and the signals are encouraging. This section includes information for REACH 2019, but it must be remembered that projects funded by these sites are only 1 year post-award.

**Table 3.** Summary technology development outcomes by program and site, as of May 1, 2021

Program	NCAI				REACH 2015				REACH 2019	Combined NCAI and REACH
Site	B-BIC	NCAI-CC	UC CAI	Total	LIBH	MN-REACH	UofL - ExCITE	Total	All sites	
Year started	2014	2014	2014		2015	2016	2016		2020	
Total projects	56	73	56	185	61	41	25	127	54	366
Investigators trained	616	202	427	1,245	600	284	129	1,717	784	3746
Follow-on funding	$218.4 M	$1331.3 M	$54.5 M	$1604.2 M	$64.8 M	$1.5 M	$7.8 M	$74.1 M	0	$1678.3 M
Start-ups	13	26	15	54	11	11	5	27	7	88
Licensed technologies	1	4	4	9	1	3	2	7	1	17
Optioned technologies	2	3	0	5	2	4	2	8	1	14
SBIR applications	14	19	3	36	4	2	6	12	1	49
STTR applications	3	12	1	16	11	3	3	17	4	37
SBIR awards	10	13	0	23	3	0	1	4	0	27
STTR awards	0	2	0	2	7	0	0	7	0	9
Technologies in clinical testing	1	9	1	11	0	1	0	1	0	12
Technologies in the market	0	2	0	2	0	1	0	1	0	3

Technologies remain under development and the outcomes presented herein are those as of May 2021. New projects are launched on a regular basis. Data updated at least semiannually.

B-BIC, Boston Biomedical Innovation Center; LIBH, Long Island Bioscience Hub; MN-REACH, Minnesota REACH; NCAI, NIH Centers for Accelerated Innovations; NCAI-CC, NIH Center for Accelerated Innovation at the Cleveland Clinic; REACH, Research Evaluation and Commercialization Hubs; SBIR, Small Business Innovation Research; STTR, Small Business Technology Transfer; UC CAI, University of California Center for Accelerated Innovation; UofL - ExCITE, University of Louisville Expediting Commercialization, Innovation, Translation, and Entrepreneurship.

The amount of follow-on funding provides a signal that outside entities perceive value in NCAI and REACH technologies and have demonstrated an interest in supporting their continued development. The total amount of follow-on funding from outside sources following the notice of the NCAI or REACH award is $1.7 billion.

This large amount of follow-on funding is not distributed equally across projects. Older projects that have had more time to mature have progressed further and attracted more interest. A couple examples from the earliest NCAI projects illustrate this point well. Dr Yogen Saunthararajah’s technology, oral THU-decitabine, a novel noncytotoxic epigenetic therapeutic, received a $400 million investment from strategic partner Novo Nordisk, which included payments tied to development and sales milestones [18]. Dr Sanford Markowitz’s technology, a small molecule inhibitor of 15PGDH, received $55 million upfront from Amgen and another $666 million is contingent on achievement of milestones [19]. These significant follow-on funding events occurred well after the projects ended and after additional technology development work had been completed.

A total of 100 NCAI projects and 52 REACH projects have received follow-on funding (as of May 2021). The single largest source of funding is corporate partners ($1.2 billion), followed by venture capital ($221 million). Foundations, associations, nonprofits, and other nonfederal grants have also been a significant source of follow-on funding ($36.3 million). Federal funders provided the majority of the balance, including SBIR and STTR awards. Twelve projects have received follow-on funding of at least $10 million.

Fifty-nine NCAI and 37 REACH projects led to 88 start-up companies. This level of start-up activity directly suggests advancement in technology readiness and advancement toward commercialization. Although start-ups are not a direct measurement of commercial readiness, the beliefs of the funded investigators and project managers about technologies’ commercial potential is demonstrated by the action to undertake the nontrivial costs and time investments associated with forming a start-up company. In total, 53 jobs have been created by 10 well-capitalized start-up companies formed around NCAI technologies.

For technologies that have not formed a start-up to further develop the technology, the execution of licensing and option agreements provides a signal of technology readiness and commercial viability as assessed by outside companies. As of May 2021, 9 NCAI and 9 REACH projects have led to 17 licensed technologies and 6 NCAI and 11 REACH projects are associated with 14 technologies with an active option agreement.^7^

In total, NCAI has produced at least 52 SBIR and STTR applications and 25 awards, and REACH has produced at least 34 SBIR and STTR applications and 11 awards. This means that the overall success rate for SBIR/STTR applications so far for the two programs is about 42%, which includes recent submissions that have not yet been evaluated for funding. Even conservatively counting the recent submissions, the SBIR/STTR application success rate among the NCAI and REACH technologies is notably greater than the approximate 19% success rate for SBIR/STTR among all applications nationwide [20]. Although award decision factors and scores are not released publicly, the sites are selecting and providing support for promising technologies, including advice about which studies to conduct to add the most value and the best technical and commercial pathways. Investigators are also receiving training about product development and how to communicate their technologies overall value proposition. Thus, one would expect that applications for SBIR/STTR awards would outperform typical success rates.

Several projects have received multiple SBIR/STTR awards. Dr Umut Gurkan’s HemeChip point-of-care sickle cell disease diagnosis technology has two SBIR and two STTR awards. Dr Jonathan Thon’s technology, a biomimetic human platelet bioreactor, Dr Daniel Lawrence’s technology, a small molecule therapeutic for the treatment of idiopathic pulmonary fibrosis, and Dr Warren Zapol’s technology, electric nitric oxide generation for medical purposes, have received a combined 11 SBIR awards. These projects have made significant progress toward the market.

NCAI and REACH technologies supported between 2014 and 2017 are beginning to reach patients. Eleven NCAI technologies and one REACH technology are in clinical testing, and two NCAI technologies and one REACH technology have been approved for marketing in the United States or abroad. Dr Thomas Gildea’s technology, custom patient-specific airway stents, was cleared by the FDA in January of 2020. More than 20 stents have been placed in patients across six centers in the United States [21]. Dr Gurkan’s HemeChip technology has obtained regulatory approvals in Africa and India and is available in nine countries at the time of writing [22]. With the significant events of 2020, a pilot version of Dr Tai Mendenhall’s self-care app for preventing compassion fatigue in disaster responders was released in all 50 states. The app is receiving national interest, especially among first responders [23].

Among the 12 technologies in clinical testing is Dr Zapol’s technology, which received emergency approval investigational device exemption in Fall 2020 to treat COVID-19. Dr Elliot Botvinick’s continuous lactate monitoring technology completed a study in Australia and Canada involving 41 patients with Type 1 Diabetes and is preparing to initiate clinical studies in the United States with COVID-19 restrictions lifting. Dr Daniel Vallera’s trispecific NK cell engager technology received IND approval and is preparing for Phase 1 trials. Nine more technologies from the NCAI-CC have begun testing on human subjects, including Dr Dominique Durand’s oropharynx appliance for maintaining airway patency and Dr Frank Papay’s neuromodulatory implant device for the treatment of obstructive sleep apnea.

Given the long development timelines often associated with these types of technologies, the speed with which NCAI and REACH technologies are reaching patients is impressive, suggesting that the sites are selecting good technologies to support and that crowding in early development work, site team expertise, and focus is steepening technologies’ trajectory.

## Best Practices Employed by NCAI and Reach Sites

Over the past several years, we have observed several practices used by the NCAI and REACH sites that have been particularly effective in managing activities and assisting applicants and funded investigators. This section describes these practices. Note that we intentionally do not include the funding itself for proof-of-concept projects in our remarks because this element is commonplace among accelerator and proof-of-concept programs. Certainly, the fact that an funded investigator typically received between $100,000 and $200,000 (and sometimes nearly $400,000) for an early-stage technology development project is significant. However, we opt to focus on the approaches that each site implemented because these speak to the ways in which site teams can add value beyond the funding award.

### Site Team Composition

Site team leadership were often members of the academic community with a strong track record of academic entrepreneurship, good visibility among faculty and staff, and the support of their institutional leadership and technology transfer offices. Their teams included project managers with backgrounds in biomedical product development, translational research, and research operations (e.g., finance, administration). Teams’ communication and interpersonal skills were especially important, particularly for working with funded investigators and helping these investigators navigate challenges and issues with their projects and identify the next steps in go-to-market strategies.

Multiple teams included representatives or leaders from their technology transfer offices and/or innovation management groups on their teams. This helped with leveraging institutional expertise, connecting investigators to other resources and programs, and developing business strategies. Teams also leveraged educational resources from their CTSAs and local innovation ecosystems.

### External Advisory Boards

External advisory boards composed of individuals from the private sector with backgrounds in science and business, investment, marketing, and/or business development are valuable for ensuring that application reviews are balanced sufficiently between a proposal’s scientific merits and the market viability of the concept. A balanced board of executives and senior researchers from life sciences companies, entrepreneurs who have brought technologies to market, and business development and commercialization experts appears to be useful to support proposal selection, advising on the site’s overall direction, and providing *ad hoc* expertise to funded investigators. Sites that began with boards principally composed of university faculty and executives often later transitioned to the aforementioned composition.

Although initiated at the program level and not the site level, the TGC was cited by many site teams and funded investigators as a valuable mechanism for early feedback and that having such feedback was validating for individual technologies in the eyes of investors and sources of follow-on funding.

### Outreach to the Investigator Community

Active outreach to the research community is important for building early awareness and interest before, during, and after solicitation cycles. During our interviews with funded investigators, many described that they were motivated to apply to NCAI or REACH after hearing directly from site personnel or faculty members who had been successful in commercialization endeavors. Effective outreach strategies include presentations at faculty meetings, promoting the mechanisms through which investigators can learn more about site funding opportunities and programs, encouraging referrals, sharing information and examples, and engaging in Q&A.

There is also a signal that the diversity of site personnel matters in cultivating a diverse applicant pool. At the University of Louisville ExCITE, investigator-facing team members, including the team lead (principal investigator), technology transfer office representative, and project managers were all women. Ultimately 63% of funded PIs or co-PIs in the program were also women [24], which was far greater than for other sites. In interviews, ExCITE investigators who were women commented that they were inspired and motivated after hearing directly from another woman faculty member and entrepreneur, Dr Paula Bates, about the program and the process of moving discoveries into the product development pipeline.

### Application Processes

In addition to scientific merit, a common application used by all sites required applicants to describe the technology’s value proposition, assess the market and competitive landscape for the technology, and provide other information about the technology’s commercial viability (e.g., intellectual property, regulatory issues). For many investigators, these application elements are unfamiliar. In response, sites’ project managers and leads often provided informal feedback or answered questions, held office hours, timed commercialization seminars such that they coincided with application cycles, and used letters of intent to provide formal feedback at earlier stages.

Many sites integrated entrepreneurial training for these elements into their pre-application assistance services, which provided additional motivation and experiential learning for investigators. They also connected applicants to reference librarians and other resources that could be useful for learning about the potential market for the technologies.

Some sites also provided small pilot awards (often $50,000 or less) to allow investigation of a technologies viability, and if successful permitted reapplication for typical award sizes. This approach was used primarily by B-BIC, which had two awards, Pilot and Drive. The approach was effective for supporting promising projects for which very limited data were available.

### Project Management

REACH and NCAI required milestone-based project management support for funded projects and project managers were part of the site teams. This function was important for monitoring progress, ensuring focus, helping address technical and business challenges, and advising on next steps. In the absence of this support, it was more likely that the investigator could drift into hypothesis-driven research and away from the validation or prototyping work funded by the programs. Tools used included target product profiles, product development plans, risk registers, and Gantt charts describing milestones and critical paths, among other tools. Project managers were also helpful for identifying when projects needed to pivot in light of market changes or research results.

Sites used a fail-fast model in which projects that did not meet milestones or generated results that indicated the project was no longer viable were terminated early. Funds were repurposed for other projects. For one project cycle, ExCITE intentionally overallocated its funding to projects, released funding in tranches, and then provided the balance of funding to those projects that demonstrated the most progress and promise.

NCAI has more funding than REACH, and these sites were generally able to support project managers with extensive private-sector product development experience with life sciences companies. Investigators prized these managers’ scientific and commercial acumen and the perspective and value they brought to the projects. Multiple NCAI-funded investigators told us that their project managers were as valuable to their projects as the funding awards themselves. REACH project managers were also effective in their roles and received praise from the investigators they supported, but with less funding available, they were less likely to have private-sector experience and often supported multiple programs.

### Skills Development Programs

More than 3700 academic investigators have participated in NCAI and REACH skills development programs to-date. Sites largely leveraged or contributed to skills development programs and offerings that were available via their Clinical and Translational Science Awards, National Science Foundation’s I-Corps Program (adapted for life scientists and investigators with clinical and research roles), or others. They focused their original content in the form of bootcamps with curricula aligned with the funding application elements. The most important mentoring was often the engagement between site teams and applicants and funded investigators about their ideas and projects. Many investigators told us that their project manager or site lead were their most important coaches and mentors and the ones from whom they learned the most about commercialization and product development.

## Concluding Remarks

To date, the NCAI and REACH programs have been effective at supporting innovators in moving promising discoveries into the product development pipeline. In less than 7 years – about a decade less than the average lag time from discovery to commercialization – three products are on the market and 12 technologies are in clinical testing. While there is significant variation in time from discovery to commercialization across individual technologies, it is uncommon for a discovery to transition to clinical practice in fewer than 10 years [25,26]. The technologies have also collectively attracted nearly $1.7 billion in follow-on investment and led to 88 start-up companies, as of May 2021.

One common measure of the effectiveness of an accelerator or proof-of-concept program is for a funder to compare its total investment to-date (which in this case is inclusive of all federal costs for completed projects plus site operating costs to-date) to the total amount of follow-on funding attracted (in this case, nonfederal funding). Currently this value is estimated to be greater than a 35× return, and north of 45× for the NCAI program because of large follow-on investments that were made in a couple of the earliest NCAI-CC-supported technologies. Removing these projects brings the outcome measures for B-BIC and NCAI-CC into alignment, demonstrating the outside impact one or two technologies can have on the total return on a portfolio.

In reviewing the results from different sites, there are several important things to keep in mind. First, underlying conditions that will affect observed outcomes include institution-specific factors, such as an institution’s research portfolio size and composition, infrastructure and innovation support programs, experience with entrepreneurship, culture, and regional innovation ecosystems. Second, NCAI and REACH had very different award parameters. NCAI sites had a longer period of performance, more funding, and were able to offer each funded project greater levels of support overall. This may affect the propensity of a researcher to submit an application for support, given their perception of the level of support available. Outcomes will also depend on the market for different technologies and therapeutic areas and differences in commercial pathways. Strong follow-on funding, for example, must be weighed against start-up creation and licensing. Lastly, none of these measures reflect the public health impact the technologies deliver, which will be important to consider as more technologies reach the market.

The NCAI and REACH programs are still operating and supporting projects. Although NCAI is set to sunset in 2022, longitudinal data collection will continue as funded technologies progress to market. More information about the results of these programs will be made available in the coming years. Few programs have provided robust information about program performance over time, and our team will be providing information about the results from NCAI, REACH 2015, and REACH 2019 in the coming years to assist academic innovation programs with calibrating their performance expectations and ensuring they have access to insights and lessons learned from these important NIH programs.

## Footnotes

1 See NIH Funding Announcements RFA-HL-13-008, RFA-OD-14-005, and RFA-OD-19-014 for further detail.

2 The TGC was originally referred to as the Technology Review Committee (TRC).

3 In addition, sites in NCAI and REACH also received multiple funding supplements to support specific NIH initiatives and priorities over the course of their award period.

4 The official website for the new Catalyze program is https://www.nhlbicatalyze.org/.

5 A description of this evaluation and data collection approach were submitted to RTI’s Institutional Review Board, which determined that the evaluation did not constitute human subject research and declined to review.

6 Technology readiness levels refer to technology readiness levels that characterize the stage of technology development, from basic research through clinical studies. More information about the nine TRL stages is available at https://www.medicalcountermeasures.gov/trl/about-the-trls//

7 There can be a many-to-one relationship between projects and technologies. For example, a site may have awarded a small pilot project and then a full project after successful pilot results.
